# Deep Fungal Infection of the Skin with Two Rare Fungi in a Dog Being Treated with Immunosuppressant Therapy: A Case Report

**DOI:** 10.3390/vetsci12100958

**Published:** 2025-10-08

**Authors:** Aimara Bello Suarez-Kupka, Sarah Ehling, Ute Siesenop, Jutta Verspohl, Andrea Vanessa Volk

**Affiliations:** 1 Hospital for Small Animals, University of Veterinary Medicine Hannover, Foundation, 30559 Hannover, Germany; sara.ehling@tiho-hannover.de (S.E.); andrea.volk@tiho-hannover.de (A.V.V.); 2 Institute of Microbiology, University of Veterinary Medicine Hannover, Foundation, 30559 Hannover, Germany; ute.siesenop@tiho-hannover.de (U.S.); jutta.verspohl@tiho-hannover.de (J.V.)

**Keywords:** fungal, infections, skin, ciclosporin, glucocorticoid, dog, treatment

## Abstract

**Simple Summary:**

An 8-year-old female intact Elo, diagnosed with immune-mediated brain inflammation, is put on immunosuppressive drugs long-term. After 48 weeks of therapy, the dog developed skin lesions on the tail and nasal cavity. This case report presents a serious mixed deep fungal skin infection with two different rare fungi on the same dog, the first report to do so in veterinary medicine. Diagnosing these infections was challenging and required tissue sampling, microscopic examination, and advanced laboratory testing to identify the fungi. Treatment involved a combination of oral antifungal medication as well as ointments, while carefully reducing immunosuppressing drugs to help the body fight the infection without worsening the original disease. Nevertheless, the dog had to be euthanised due to the worsening of the underlying disease. This case report highlights the importance of regular dermatological and clinical checks, early testing of suspicious lesions, and tailored treatment plans for pets on immunosuppressive therapy. Better monitoring of skin lesions and health status could lead to earlier diagnosis and better outcomes, which would benefit animal health and guide veterinarians and animal owners for future action.

**Abstract:**

Deep fungal skin infections in dogs being treated with immunosuppressant therapy pose a clinical challenge, balancing effective antifungal therapy with a sufficient immune function to control the infection whilst maintaining good control over the original disease. This case report describes the clinical presentation, diagnosis, and outcome of a dog with a concurrent deep fungal infection of the skin with two rare fungi, *Purpureocillium sodanum* and *Alternaria rosae*, and this is the first time this agents has been described in veterinary medicine. Challenges included fungal infections at multiple sites and drug–drug interactions between necessary immunosuppressive therapy and antifungals. Persistent lesions and limited therapeutic success emphasise the need for clinicians’ awareness, early diagnosis, individualised case management, and advances in immunosuppressive protocols.

## 1. Background

Meningoencephalitis of unknown origin (MUO) relies on immunosuppressive therapy, with glucocorticoids being the cornerstone of treatment due to their effects on the humoral and cell-mediated immune response [1,2,3]. Different treatment protocols have been suggested to reduce the glucocorticoid dose and avoid severe steroid-related side effects. Glucocorticoids alone, or in combination with other drugs, are currently the mainstay of treatment for dogs suffering from MUO [1].

With this in mind, a number of second-line drugs have found their way into the routine treatment of dogs with MUO, including ciclosporin, mycophenolate mofetil, cytosine arabinoside, lomustine, leflunomide, and azathioprine [1].

Ciclosporin is a reasonable and widely used add-on to prednisolone for MUO due to its potent immunosuppressive features without suppressing the bone marrow [1,3]. It is a calcineurin inhibitor that blocks the activation of T-cells, thus preventing the synthesis of various cytokines, in particular interleukin 2 [4], and, in combination with glucocorticoids, it is a defensible first-line or early combination for many MUO dogs, with evidence of long survivals and reduced relapse rate [2,3,5]. Due to their mechanism of action, patients have a higher tendency to develop secondary infections aside from other side effects, including fungal infections. Those might manifest from localised cutaneous fungal infections to systemic mycoses, particularly of the respiratory and urinary tracts [6,7,8,9,10]. In the case series by Dowling et al., the prevalence of fungal infections was described as 1.67% with the drug combination of prednisolone and ciclosporin [8].

Secondary fungal infections in immunocompromised dogs have been described in the literature with dematiaceous moulds, such as *Curvularia* spp., *Alternaria* spp., *Phialophora* spp., *Trematospheria* spp., and *Paecilomyces* spp., as well as hyaline moulds such as *Aspergillus citrinoterreus*, *Fusarium* spp., and *Byssochamys* spp. [11,12,13]. The risk of infection is higher the longer the treatment course of combination therapy with prednisolone and ciclosporin, and/or the higher the doses used. Doses of 5–6 mg/kg twice daily for ciclosporin and 0.5–2 mg/kg daily for glucocorticoids are reported in the literature at the time of diagnosis of fungal infections [7,8].

To our knowledge, this case report describes for the first time a mixed deep fungal infection of the skin in a dog with *Purpureocillium sodanum* and *Alternaria rosae* in the course of immunosuppressive therapy. The diagnostic complexity, the therapeutic challenges, and the importance of individualised tapering of immunosuppressive drugs are emphasised.

## 2. Case Presentation

**Figure 1:**
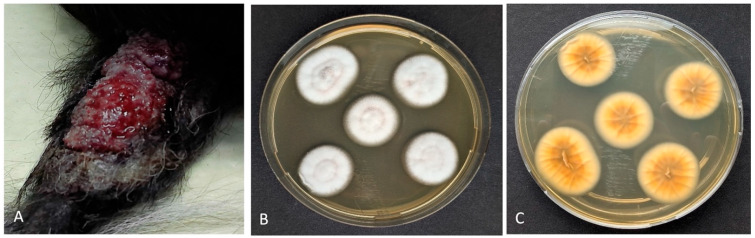
Canine deep fungal infection: (**A**) Papular plaque with ulcerated surface on the dorsoproximal aspect of its tail. (**B**) Isolation of *Purpureocillium* sp. (via macerated tissue culture). Characteristic colonies with a velvety to powdery surface and a light purple pigmentation on the underside (**C**).

**Figure 2:**
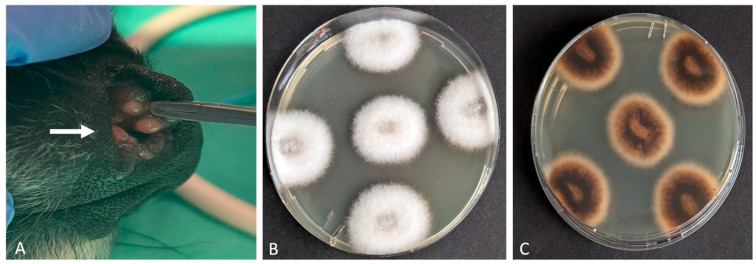
Canine deep fungal infection: (**A**) Multiple papular and nodular intranasal lesions (white arrow). Isolation of *Alternaria rosae* (via macerated tissue culture). Fungal colonies with a white, cotton-like surface (**B**) and a yellow-brown reverse pigmentation on the underside (**C**).

An 8-year-old female intact Elo diagnosed with meningoencephalitis of unknown origin (MUO) was treated with prednisolone (maintenance dose of 0.5 mg/kg, once daily) and ciclosporin (maintenance dose of 6.8 mg/kg, twice daily). After 48 weeks of therapy, the dog developed ulcerative dermatitis on the tail (Figure 1) and papular to nodular lesions in the nasal cavity four weeks later (Figure 2).

**Figure 3:**
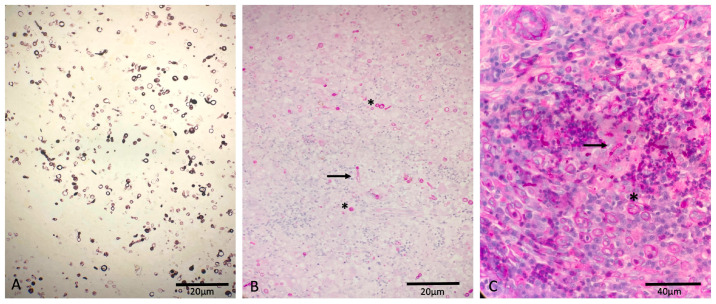
Canine deep fungal infection: (**A**) Grocott-Silver positive-stained conidia from the nasal tissue. (**B**) PAS-positive-stained conidia (asterisk) and hyphae (arrow) from the nasal tissue. (**C**) PAS-positive-stained conidia (asterisk) and hyphae (arrow) from the tail tissue.

Cytology of the surface impression and fine needle aspiration of the lesions revealed fungal hyphae. On histology, severe pyogranulomatous deep dermatitis with intralesional conidia, positive on PAS- and Grocott-Silver-special staining, was observed from the nasal tissue and septated hyphae from the tail tissue (Figure 3).

Microbiological cultures (from macerated tissue), performed at the Institute of Microbiology of the University of Veterinary Medicine Hannover, and PCR of ITS (Internal Transcribed Spacer) regions performed at the National Reference Centre for Invasive Fungal Infections (NRZMyk), revealed *Purpureocillium sodanum* in the tail lesions and *Alternaria rosae* in the nasal lesions of the same dog. A susceptibility test was performed using the EUCAST microdilution test for *Purpureocillium sodanum* and for *Alternaria rosae*. *Purpureocillium sodanum* showed the following MIC: Amphotericin B (MIC = >16 µg/mL), voriconazole (MIC = 0.25 µg/mL), anidulafungin (MIC = 0.06 µg/mL), posaconazole (MIC = 0.06 µg/mL), and itraconazole (MIC = 2 µg/mL). *Alternaria rosae* showed the following MIC: Posaconazole (MIC = 0.25 µg/mL), voriconazole (MIC = 2 µg/mL), itraconazole (MIC = 8 µg/mL), and isavuconazole (MIC = 4 µg/mL).

For the assessment of possible ascending opportunistic fungal infections of the urinary tract, a cystocentesis sample was submitted for fungal culture, revealing no fungal organisms. Bacterial assessment of the same urine sample revealed bacterial cystitis with *Escherichia coli*, which was treated orally twice daily with amoxicillin and clavulanic acid, according to sensitivity results. Computer tomography (CT) of the head, thorax, and abdomen showed no evidence of fungal invasion.

Despite systemic therapy with itraconazole (5 mg/kg once daily) and topical antifungal therapy with posaconazole for the tail lesions and terbinafine for the nasal lesions, as well as a 50% dose reduction in ciclosporin (to 6.8 mg/kg once daily) to avoid overdosing when combined with itraconazole, the fungal lesions did not regress. On the other hand, the dog’s neurological condition worsened despite an increase in the prednisolone dose to keep the underlying disease under control, so that she had to be euthanised due to the severe deterioration in her quality of life.

## 3. Discussion

This case report describes a deep opportunistic fungal infection in the skin of a dog whilst receiving immunosuppressive therapy, highlighting the need for clinicians’ awareness, early diagnosis, individualised case management, and advances in immunosuppressive protocols in cases of persistent lesions and limited therapeutic success in clearing the infection. In addition, this is the first report of a co-infection with *Purpureocillium sodanum* and *Alternaria rosae* at multiple sites in the same immunocompromised dog. In the literature, there are only a few case reports of opportunistic infections with *Purpureocillium lilacinum*, formerly known as *Paecilomyces lilacinus*, in human and veterinary medicine [14,15,16]. Albert et al. describe one case of skin infection with *Purpureocillium lilacinum* in an immunocompromised patient after renal transplantation [15]. In the feline case reports by Rosser et al. and by Pawloski et al., one cat presented with a history of recurrent soft tissue swelling in the left metacarpal region, and the other cat presented with multifocal nodules in the right caudal lung lobe parenchyma [14,16]. In humans, *Alternaria* species can present as sinusitis, pneumonia, or wound infections following surgery, and the onset or significant exacerbation of alternariosis has been described following corticosteroid therapy [17]. In 2023, Blaga et al. presented one case of simultaneous infection of *Toxoplasma gondii* and *Alternaria* spp. in a dog with nodular skin lesions on the thorax and on the nasal bridge, which was previously treated with ciclosporin and prednisolone for immune-mediated haemolytic anaemia IMHA [18].

Fungal infections are frequently underestimated complications in dogs undergoing immunosuppressive therapy [7,8,9]. Ciclosporin and glucocorticoids impair T-cell function and cytokine release, resulting in ineffective fungal clearance [7,8,9]. This predisposes dogs to deep tissue- and disseminated fungal infections [9,19].

Previous studies confirm an increased risk of fungal infections in dogs receiving high immunosuppressive doses of ciclosporin in the range of 8.8–17 mg/kg once daily [7,8,9]. In one study, 13% of ciclosporin-treated dogs developed opportunistic fungal infections, some of which led to systemic disease [7]. Similarly, Dowling et al. found that opportunistic fungal infections typically occur 4–6 weeks after starting ciclosporin treatment, possibly due to a pre-existing fungal colonisation [8]. In this case report, the fungal infections were detected after a period of 48 weeks, possibly making a pre-existing fungal colonisation less likely, and a secondary infection related to the weakened immune system more likely. Furthermore, the doses of prednisolone and ciclosporin in our case were 0.5 mg/kg once daily for prednisolone and 6.8 mg/kg twice daily for ciclosporin, confirming that secondary fungal infections can be diagnosed in dogs treated with anti-inflammatory doses of glucocorticoids when combined with higher doses of ciclosporin in comparison to previous reports.

The localisation of the skin lesions is of special interest in this case report, suggesting that a possible portal of entry for this organism could be the upper respiratory tract, as well as wounds or micro-injuries during walks, as widely reported in other cases of fungal infections [8,9,11,20]. This underlines the importance of routine dermatological but also systemic monitoring, including urinalysis and the offer of diagnostic imaging (e.g., X-rays or CT), of, e.g., the head, thorax, or whole body, to owners of patients receiving long-term combination therapy of prednisolone and ciclosporin. If the owner declines further investigations, the physician should consider reducing the immunosuppressive therapy until the skin lesions are under control. In our case, a reduction in the ciclosporin dose by half during antifungal therapy was recommended, predominantly to avoid interactions between ciclosporin and itraconazole [21], but also to allow clearance of the infection.

In general, the treatment of fungal infections in immunocompromised dogs requires a balance between antifungal medication to clear the infection and sufficient immunosuppression to control immune-mediated disease. Due to its broad spectrum activity and safety, itraconazole is the treatment of choice for non-life-threatening systemic mycoses that do not affect the central nervous system, e.g., *Aspergillosis*, *Blastomycosis*, *Candidiasis*, *Cryptococcosis*, *Dermatophytosis*, *Histoplamosis*, *Malassezia* spp., and *Sporotrichosis* in dogs [22,23]. Itraconazole has been widely used in the treatment and prophylaxis of fungal infections in human and veterinary medicine, with treatment duration extending over several months, depending on clinical response [8,9,19]. However, the selection of appropriate treatment depends on the localisation and extent of the infection, as well as the sensitivity testing (if agreed by owners), efficacy, safety profile, and pharmacokinetics of the available drugs [24].

For the treatment of mould infections, the European Confederation of Medical Mycology (ECMM) together with the International Society for Human and Animal Mycology (ISHAM) and the American Society for Microbiology (ASM) has produced a comprehensive guideline as part of its One World—One Guideline initiative to facilitate clinical decision-making while providing an overview of the areas of uncertainty in invasive mould infections [25].

Various laboratory methods are available to evaluate or screen the in vitro antimicrobial activity of an extract or pure compound. Among these, the broth microdilution method is considered the gold standard for antifungal susceptibility testing, as endorsed by both the European Committee on Antimicrobial Susceptibility Testing (EUCAST) and the Clinical and Laboratory Standards Institute (CLSI) [26].

These organisations have set limits for certain antifungal agents against *Candida* spp. and *Aspergillus* spp. Both EUCAST and CLSI provide clinical breakpoints for common *Candida* spp. and antifungals, but there are gaps for rare species and certain drug classes. EUCAST generally has no breakpoints for *Aspergillus* spp. and instead uses epidemiological cut-off values (ECOFFs) [27]. CSLI also lacks *Aspergillus* spp. breakpoints and has species-specific gaps [27,28]. In our case, a susceptibility test using the EUCAST microdilution test for *Purpureocillium sodanum* showed a remarkably high MIC for amphotericin B (>16 µg/mL), indicating strong resistance. This is consistent with published data for *Purpureocillium lilacinum*, which is known to have intrinsic resistance to polyenes [15]. Azole antifungals showed variable activity. In our case, posaconazole and voriconazole showed the most promising results (MIC = 0.06 µg/mL and 0.25 µg/mL, respectively), supporting their potential use as primary agents. Itraconazole, on the other hand, showed a relatively high MIC (2 µg/mL), suggesting reduced efficacy, particularly in systemic infections where adequate drug concentrations may be difficult to achieve. In our case, however, no systemic infection was confirmed, so a combined therapy with topical posaconazole and systemic itraconazole was chosen. In *Alternaria rosae*, posaconazole showed the most favourable MIC (0.25 µg/mL), followed by voriconazole (MIC = 2 µg/mL). In contrast, itraconazole and isavuconazole showed higher MIC values (MIC = 8 µg/mL and 4 µg/mL, respectively), suggesting lower susceptibility. These results are consistent with the published literature, which indicates that *Alternaria* spp. often exhibit variable susceptibility to azoles, necessitating species-specific and case-dependent therapy [29]. A wide MIC range for itraconazole, voriconazole, and posaconzole in *Alternaria* spp. and isolated species is reported in the published data.

Although the EUCAST breakpoints are widely recognised as a guideline for antifungal therapy, it is important to emphasise that these clinical breakpoints were developed specifically for *Candida* spp. and *Aspergillus* spp. [25]. The MIC values observed in this case provide a useful comparative framework, but they must be interpreted with caution, and the data cannot be directly translated into clinical treatment decisions. Therapeutic decisions should take into account in vitro data, pharmacodynamic and pharmacochemical properties, drug approval, clinical experience, and host immune status [25].

A key challenge in this patient was to adjust the immunosuppressive therapy whilst avoiding relapse of the immune-mediated disease. Ideally, immunosuppressive therapy should be discontinued, but this is not always possible and was not possible in our case due to the life-threatening nature of the underlying disease. In our case, the ciclosporin doses were reduced to allow the immune system to better clear the fungal infection without completely stopping the immunosuppressants, and, additionally, to avoid drug interactions with itraconazole.

A number of drug interactions, including azoles, compete with the hepatic P-450 enzyme system, leading to increased blood concentrations of ciclosporin due to reduced clearance [21]. In our case, reducing the dose of ciclosporin by half rather than abruptly discontinuing it has also made it possible to recognise a relapse of the underlying disease.

To summarise, secondary deep cutaneous fungal infections are a significant risk in immunocompromised dogs, particularly those receiving ciclosporin and glucocorticoids in combination.

This case report presents new insights into cutaneous fungal infections with multiple pathogens in a dog, including identification via sequencing of ITS regions and susceptibility testing of new fungi, as well as the dosing regimens of ciclosporin and glucocorticoids with the exact timing of first appearance of skin lesions after initiation of immunosuppressive therapy.

We hereby like to highlight the need for surveillance of immunocompromised high-risk patients regarding deep cutaneous fungal infections, including skin surface cytology in erosive or ulcerative lesions, FNA or biopsies for nodular lesions, urinalysis, imaging, and, in positively diagnosed cases, targeted antifungal therapy and careful adjustment of immunosuppressive medications to optimise outcomes.

## 4. Conclusions

This case report describes a mixed multisite deep fungal infection of the skin in an immunosuppressed dog. Our findings highlight the therapeutic challenges of treating secondary fungal infections while controlling autoimmune disease and argue for multidisciplinary collaboration, a combined diagnostic approach (microscopy, culture, and molecular/antigenic methods), and individualised strategies to reduce immunosuppressive drugs. Future research should investigate larger prospective studies and therapeutic drug monitoring to optimise antifungal strategies in these patients.

## Data Availability

The original contributions presented in this study are included in the article. Further inquiries can be directed to the corresponding author.

## Abbreviations

The following abbreviations are used in this manuscript:

MUO	Meningoencephalitis of Unknown Origin
PCR	Polymerase Chain Reaction
ITS	Internal Transcribed Spacer
EUCAST	European Confederation of Medical Mycology
MIC	Minimum Inhibitory Concentration
CT	Computed Tomography
ECMM	European Confederation of Medical Mycology
ISHAM	Internal Society for Human and Animal Mycology
ASM	American Society for Microbiology
CLSI	Clinical and Laboratory Standards Institute
ECOFF	Epidemiological Cut-Off Value
